# Differentiation of retroperitoneal paragangliomas and schwannomas based on computed tomography radiomics

**DOI:** 10.1038/s41598-023-28297-6

**Published:** 2023-06-07

**Authors:** Yuntai Cao, Zhan Wang, Jialiang Ren, Wencun Liu, Huiwen Da, Xiaotong Yang, Haihua Bao

**Affiliations:** 1 Department of Radiology, Affiliated Hospital of Qinghai University, Tongren Road No.29, Xining, 810001 People’s Republic of China; 2 Department of Biomedical Engineering, Tsinghua University, Beijing, People’s Republic of China; 3 Department of Pharmaceuticals Diagnosis, GE Healthcare, Beijing, People’s Republic of China; 4 Department of Radiology, Chongqing Jiulongpo People’s Hospital, Chongqing, People’s Republic of China

**Keywords:** Endocrinology, Medical research, Oncology

## Abstract

The purpose of this study was to differentiate the retroperitoneal paragangliomas and schwannomas using computed tomography (CT) radiomics. This study included 112 patients from two centers who pathologically confirmed retroperitoneal pheochromocytomas and schwannomas and underwent preoperative CT examinations. Radiomics features of the entire primary tumor were extracted from non-contrast enhancement (NC), arterial phase (AP) and venous phase (VP) CT images. The least absolute shrinkage and selection operator method was used to screen out key radiomics signatures. Radiomics, clinical and clinical-radiomics combined models were built to differentiate the retroperitoneal paragangliomas and schwannomas. Model performance and clinical usefulness were evaluated by receiver operating characteristic curve, calibration curve and decision curve. In addition, we compared the diagnostic accuracy of radiomics, clinical and clinical-radiomics combined models with radiologists for pheochromocytomas and schwannomas in the same set of data. Three NC, 4 AP, and 3 VP radiomics features were retained as the final radiomics signatures for differentiating the paragangliomas and schwannomas. The CT characteristics CT attenuation value of NC and the enhancement magnitude at AP and VP were found to be significantly different statistically (*P* < 0.05). The NC, AP, VP, Radiomics and clinical models had encouraging discriminative performance. The clinical-radiomics combined model that combined radiomics signatures and clinical characteristics showed excellent performance, with an area under curve (AUC) values were 0.984 (95% CI 0.952–1.000) in the training cohort, 0.955 (95% CI 0.864–1.000) in the internal validation cohort and 0.871 (95% CI 0.710–1.000) in the external validation cohort. The accuracy, sensitivity and specificity were 0.984, 0.970 and 1.000 in the training cohort, 0.960, 1.000 and 0.917 in the internal validation cohort and 0.917, 0.923 and 0.818 in the external validation cohort, respectively. Additionally, AP, VP, Radiomics, clinical and clinical-radiomics combined models had a higher diagnostic accuracy for pheochromocytomas and schwannomas than the two radiologists. Our study demonstrated the CT-based radiomics models has promising performance in differentiating the paragangliomas and schwannomas.

## Introduction

Pheochromocytomas and paragangliomas are neuroendocrine tumors that originate from chromaffin cells of the adrenal medulla and extra-adrenal paraganglia^1^. In the abdominal cavity, paragangliomas (extra-adrenal pheochromocytomas) mainly occur in the retroperitoneum^2,3^. Retroperitoneal extra-adrenal paragangliomas account for 1–3% of all retroperitoneal tumors^3^. Retroperitoneal paragangliomas can be divided into functional paragangliomas and nonfunctional paragangliomas; functional tumors are often accompanied by hypertension, tachycardia, headache and diuretic symptoms^4^. Nonfunctional paragangliomas are typically asymptomatic with normal catecholamine levels^5^. Approximately half of all retroperitoneal paragangliomas are nonfunctional or potentially functional^6,7^. However, if these functional and potentially functional tumors are misdiagnosed before surgery, intraoperative compression of the tumor may lead to a sudden release of catecholamines with disastrous consequences, such as tachycardia and hypertension crisis^8–10^. If pheochromocytoma is accurately diagnosed before surgery, drugs including α- and β-adrenergic receptor antagonists and calcium channel blockers and/or drugs that inhibit catecholamines synthesis may be administered preoperatively to prevent catecholamines release.

Schwannomas are benign encapsulated neoplasms formed by the peripheral nerve sheath. Schwannomas are most common in the head and neck and rarely occur in the retroperitoneum^11^. Schwannomas account for 4% of all retroperitoneal tumors^12^. Most retroperitoneal schwannomas have no clinical symptoms and are found only upon a physical examination or incidentally^13^.

Both paragangliomas and schwannomas are rare solid tumors occurring in the retroperitoneal space. Approximately half of the tumors are clinically asymptomatic and have some similar radiological features, such as cysts, necrosis, hemorrhage and calcification^14,15^, and differentiating them before surgery has always been a challenge for clinicians and radiologists. More importantly, any physical contact with paragangliomas may result in serious consequences. Therefore, it is very important to find a non-invasive, easily repeatable method to differentiate the two types of tumors. Radiomics refers to the extraction of a large number of quantitative imaging features from medical data, and evaluating its association with heterogeneity of tumor^16^. To the best of our knowledge, no study has shown the ability of a radiomics to discriminate between retroperitoneal paragangliomas and schwannomas. Therefore, this study aimed to investigate whether CT radiomics could differentiate retroperitoneal paragangliomas from schwannomas.

## Materials and methods

### Patients

**Figure 1:**
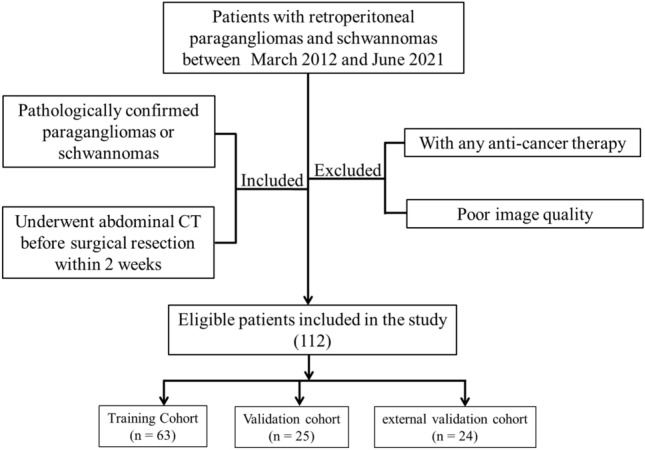
Flow diagram of the recruitment pathway.

This study retrospectively analyzed the clinical and imaging data of 112 patients with retroperitoneal paragangliomas and schwannomas confirmed by pathology between March 2012 and June 2021, 88 patients were recruited from Affiliated Hospital of Qinghai University (center I) and 24 patients were recruited from Chongqing Jiulongpo People's Hospital (center II). Inclusion criteria were as follows: (1) pathologically diagnosed paragangliomas and schwannomas; (2) contrast-enhanced abdomen CT was performed before surgery. The exclusion criteria were as follows: (1) without any prior treatment before surgical resection; and (2) poor image quality. Clinical data included age, sex, complaint and history of hypertension. Preoperative CT images were evaluated qualitatively and quantitatively. Qualitative data included the shape and margin of the tumor; the presence or absence of intratumoral hemorrhage, calcification and cysts or necrosis in the tumor. Quantitative data included the maximum diameter of the tumor, CT attenuation value of non-contrast enhancement (NC), arterial phase (AP), and venous phase (VP), and the enhancement magnitude at AP and VP. Two radiologists with more than 10 years of experience in abdominal radiology performed the image analysis. Both radiologists were blinded to the clinical and pathological data of the patients. To minimize bias, the quantitative data was taken as the final result by the average of the measurement values of the two radiologists, while the qualitative data is diagnosed by the two radiologists independently, and the disagreement is resolved through negotiation. Figure 1 shows the patient recruitment flowchart. All processes of this study conformed to the ethical standards of the institutional and national medical ethics committees, as well as to the 1964 Declaration of Helsinki and similar ethical standards. This observational study was approved by the Medical Ethics Committee of Affiliated Hospital of Qinghai University and Medical Ethics Committee of Chongqing Jiulongpo People's Hospital, and the requirement of informed consent was waived because of the retrospective nature of this study.

### CT image acquisition and segmentation

All patients underwent a multiphase contrast-enhanced CT examination before the operation. Abdominal CT scans were performed on an iCT 256 scanner (Philips, Amsterdam, Netherlands) with a tube voltage of 120 kV, a tube current of 251 mAs, a collimator thickness of 80 mm, a rotation time of 0.5 s, a screw pitch of 1.150:1 and a reconstructed layer thickness of 1.0 mm. The matrix size of CT was 512 × 512, the voxel size was 0.887 × 0.887 × 1.00 mm^3^, the field of view (FOV) was 450 mm. The scanning phase includes NC and enhancement scans. Enhanced scan was performed using a power-injector to inject intravenous iohexol (1 ml/kg) through the antecubital fossa at an injection rate of 3.5–4.5 ml/s. The AP and VP were scanned at 25–30 s and 60–70 s, respectively, after the injection of contrast.

The original images of NC, AP and VP were stored in the corresponding folders in DICOM format. One abdominal radiologist (Y T C) performed three-dimensional (3D) radiomics segmentation on NC, AP and VP images using ITK-SNAP software (version 3.6.0; www.itksnap.org). The window width and window level were seted at 40 and 300, respectively. For radiomics segmentation, the ROI was manually delineated on each slice of the tumor. Finally, each tumor generated three ROIs (NC, AP and VP).

### Feature extraction, feature selection and radiomics prediction model building

Radiomics features were extracted and selected using PyRadiomics software (version:3.0.0). The PyRadiomics parameters are as follows: binwidth 25, interpolator with Bspline, voxel resampling set as 1 × 1 × 1 mm (isotropy); LoG filter with sigma set as $\sigma =1, 3, 5 \; \mathrm{mm}$, and wavelet filter set coif1 as type of wavelet decomposition. Seven classes of radiomics features (first-order histogram, morphologic, grey level co-existence matrix (GLCM), grey level range-matrix (GLRM), grey level size zone matrix (GLSZM), neighbouring grey tone difference matrix (NGTDM) and grey level dependence matrix (GLDM) features were extracted from original and filtered images (wavelet and Laplacian of Gaussian).

**Figure 2:**
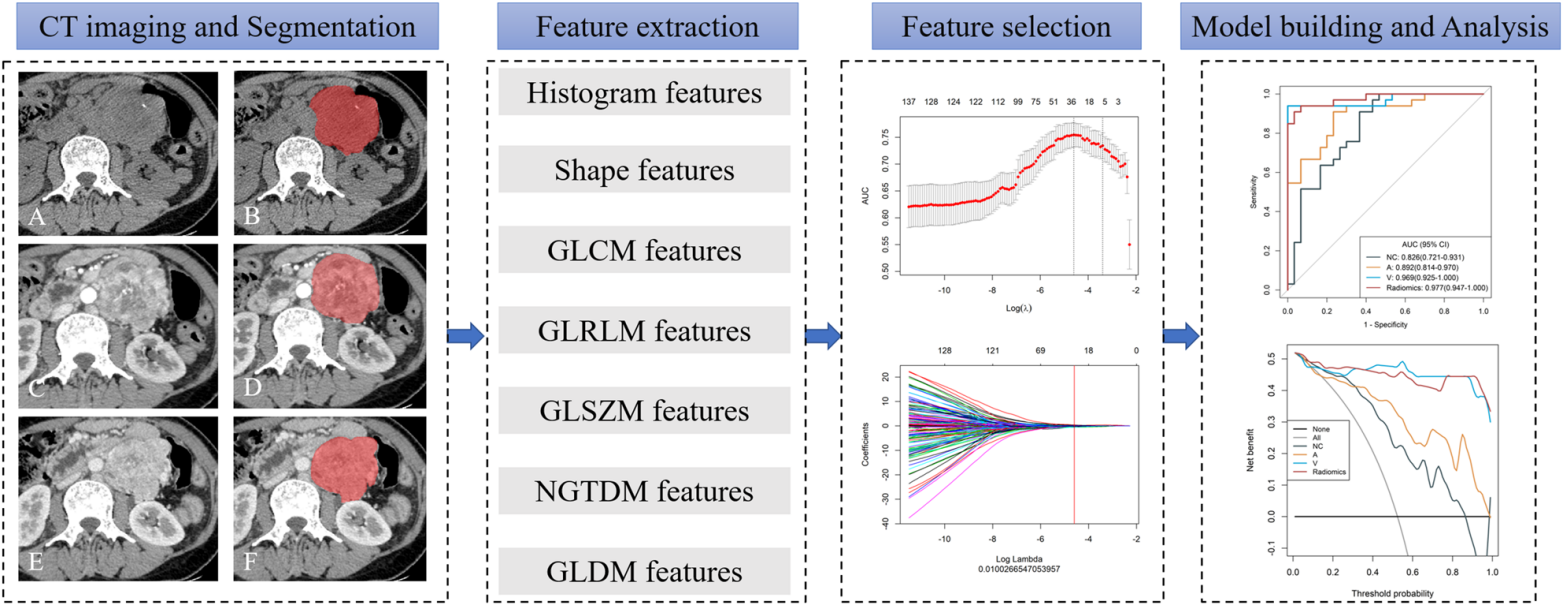
Workflow of radiomics model building and analysis. The tumors were segmented on no contrast enhancement (**A**,**B**), arterial phase (**C**,**D**) and venous phase (**E**,**F**) CT images to form volumes of interest (VOIs). One thousand and thirty-seven quantitative radiomics features were extracted from each patient. The least absolute shrinkage and selection operator (LASSO) was used to select the features. Multivariate logistic regression was used to build radiomics models for differentiating the paragangliomas and schwannomas. Receiver operating characteristic curves and decision curves were used to evaluate the clinical usefulness of the radiomics models.

After radiomics features extraction, z-score normalisation was performed on each feature. Then, the most importance features were selected to differentiate retroperitoneal paragangliomas from schwannomas using a three-step procedure. Firstly, univariate analysis was performed for feature selection to retain the feature with *P* < 0.05 to enter the following process. Secondly, the least absolute shrinkage and selection operator (LASSO) analysis was used to retain the key features for differentiating paragangliomas and schwannomas. Finally, multivariable stepwise regression further eliminates irrelevant features and retains the most informative features as radiomics signatures. The multivariate logistic regression was used to build radiomics models for differentiating paragangliomas and schwannomas. A ten times five-fold cross-validation was applied to avoid overfitting and to identify the model with the best performance. Three radiomics models were established based on the above radiomics signatures in CT images (NC, AP and VP). Further, a Radiomics combined model was built based on multivariate logistic regression method from NC, AP and VP fusion features. The workflow of radiomics model building and analysis is shown in Fig. 2.

### Clinical and combined model construction

For clinical and CT characteristics, the Chi-squared test or Fisher’s exact test were used to compare the differences in sex, hypertension, symptom, shape, margin, hemorrhage, calcification and cysts or necrosis, while the Student’s t-test or Mann–Whitney U test were used to compare the differences in age, maximum diameter, CT attenuation value of NC and the enhancement magnitude at AP and VP between paragangliomas and schwannomas. Generally, *P* values < 0.05 (two-sided) were considered statistically significant. We performed multivariable analyses to identify the most important features. A clinical model was established based on the inclusion of selected features.

A clinical-radiomics combined model was developed based on correlated clinical risk factors, correlated CT characteristics and radiomics features to verify whether the combination of radiomics signatures and clinical factors could improve performance in differentiating paragangliomas and schwannomas. The multivariate logistic regression analysis was used to construct a clinical-radiomics combined model in the training cohort, and the discrimination ability of the combined model was evaluated in the internal validation cohort and external validation cohort.

In addition, we compared the diagnostic accuracy of radiomics, clinical and clinical-radiomics combined model models with radiologists in the same set of data for pheochromocytomas and schwannomas. The abdominal CT images of pheochromocytomas and schwannomas (including NC, AP and VP imags) were analyzed by two abdominal radiologists with more than 10 years of experience in abdominal imaging diagnosis without knowledge of the pathological results, they made their decision based on analysing all 3 phases together.

### Statistical analysis

The continuous and classification variables of paragangliomas and schwannomas are represented as the mean ± standard deviation (mean ± SD) and n (%), respectively. All data analyses were performed by using the R statistical software package (version 3.6.3; http://www.Rproject.org). The Student’s t-test or Mann–Whitney U test was used to compare the continuous variables of paragangliomas and schwannomas. A chi-square test and Fisher’s exact test were used for categorical variable comparison. A *P* value < 0.05 was considered statistically significant. ROC analysis was used to evaluate the predictive accuracy of the different models. The AUC value and 95% confidence interval (CI), accuracy, sensitivity, specificity, positive predictive value (PPV) and negative predictive value (NPV) were also calculated. Comparison of the ROC curves of different models was carried out using the Delong test. A calibration curve was constructed to assess the goodness-of-fit of the models. To verify the clinical usefulness of the models, we quantified the net benefit at different threshold probabilities in the dataset using decision curve analysis (DCA).

## Results

### Clinical and CT characteristics

A total of 112 patients with 112 tumors from two centers entered the final analysis, including 59 paragangliomas and 53 schwannomas*.* Stratified sampling method was used to categorise the patients from center I into a training cohort (n = 63) and an internal validation cohort (n = 25) in the ratio of 7:3. In addition, we used 24 patients from center II as external validation cohort. The training cohort was used for model building and the internal validation cohort and external validation cohort were used for model validation. Clinical and tumor characteristics of center I are listed in Table 1. As shown in Table 1, there were 46 patients with a paraganglioma, including 16 males (34.8%) and 30 females (65.2%), with an average age of 47.70 ± 12.94 years. There were 42 patients with a schwannoma, including 18 males (42.9%) and 24 females (57.1%), with an average age of 49.88 ± 11.22 years. There were no significant differences in age and gender between the two groups (*P* > 0.05, Table 1). The prevalence of paragangliomas associated with hypertension was higher than that of schwannomas, but the difference was not statistically significant (26.1% vs. 11.9%, *P* > 0.05). Seventeen patients with a paraganglioma (37.0%) and 18 patients with a schwannoma (42.9%) were found incidentally.

**Table 1:** Clinical and CT characteristics of retroperitoneal paraganglioma and schwannoma.

Variable	Comparison group*		*P*-value
	Paraganglioma (n = 46)	Schwannoma (n = 42)	
Age (year)	47.70 ± 12.94	49.88 ± 11.22	0.402
Male, %	16 (34.8)	18(42.9)	0.437
Hypertension, %	12 (26.1)	5 (11.9)	0.092
Symptom			0.769
Asymptomatic, %	17 (37.0)	18 (42.9)	
Abdominal pain, %	15 (32.6)	15 (35.7)	
Waist pain, %	8 (17.4)	6 (14.3)	
Dizziness, vomiting, %	6 (13.0)	3 (7.1)	
Maximum diameter, mm	56.71 ± 27.81	50.16 ± 25.57	0.316
Shape, %			0.490
Circular/quasi-circular	37 (80.4)	37 (88.1)	
Irregular	9 (19.6)	5 (11.9)	
Margin, %			0.908
Well circumscribed	43 (93.5)	39 (92.9)	
Poorly circumscribed	3 (6.5)	3 (7.1)	
Calcification, %	14 (30.4)	15 (35.7)	0.765
Hemorrhage, %	12 (26.7)	7 (16.7)	0.385
Necrosis or cystic, %	35 (76.1)	30 (71.4)	0.800
Unenhanced CT value, HU	41.22 ± 8.82	35.41 ± 9.54	0.007
△Arterial phase value, HU	50.61 ± 22.82	28.16 ± 8.36	< 0.001
△Venous phase value, HU	46.90 ± 15.31	33.86 ± 8.80	< 0.001

*P*-values were calculated by Student’s t-test, Chi-square test or Fisher’s exact test between paragangliomas and schwannomas patients, where appropriate.

△Arterial phase was defined as the CT attenuation value of arterial phase minus the CT attenuation value of unenhanced CT.

△Venous phase was defined as the CT attenuation value of venous phase minus the CT attenuation value of unenhanced CT.

*Values are mean ± SD or no. (%).

### Clinical model building

**Figure 3:**
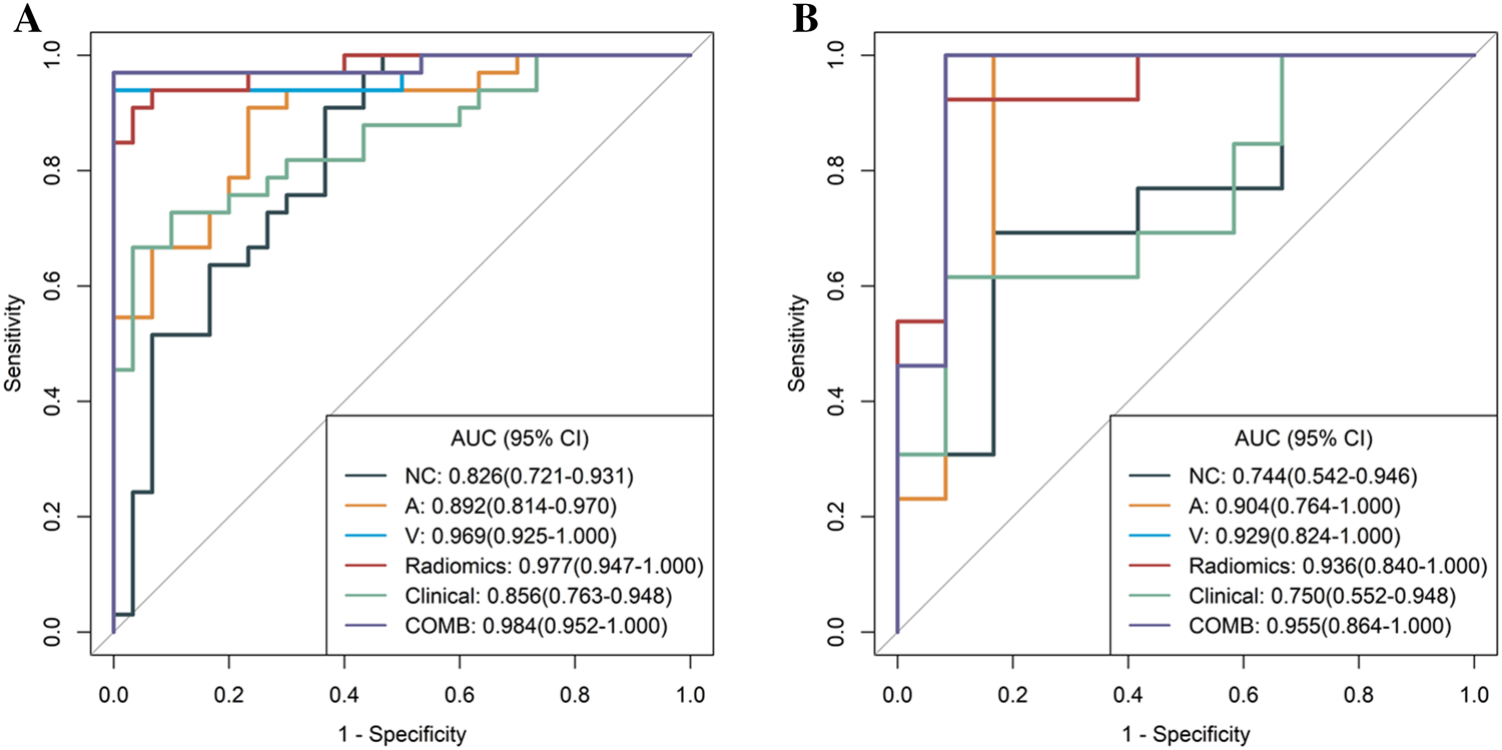
ROC curves of the different models in training (**A**) and internal validation cohorts (**B**). *A* radiomics model of arterial phase, *AUC* area under the curve, *COMB* radiomics model and clinical model, *D* radiomics model of delayed phase, *Radiomics* radiomics model of fusion of arterial phase, delayed phase and venous phase features, *V* radiomics model of venous phase.

In the training cohort, the CT characteristics CT attenuation value of NC and the enhancement magnitude at AP and VP were found to be significantly different statistically (all *P* < 0.05), and the other CT characteristics and clinical characteristics were not significantly different (all *P* > 0.05) between paragangliomas and schwannomas. After multivariate regression analyses, the enhancement magnitude at AP and VP were selected as independent predictors and enrolled into clinical model. The clinical model showed good performance for differentiating paragangliomas and schwannomas in the training cohort, internal validation cohort and the external validation cohort, with the AUC being 0.856 (95% CI 0.763–0.948) in the training cohort, 0.750 (95% CI 0.552–0.948) in the internal validation cohort and 0.787 (95% CI 0.591–0.982) in the external validation cohort (Tables 3, 4; Fig. 3). The accuracy, sensitivity, and specificity were 0.810, 0.667 and 0.967 in the training cohort, 0.760, 0.615 and 0.917 in the internal validation cohort and 0.791, 0.846 and 0.727 in the external validation cohort, respectively (Tables 3, 4; Fig. 3).

### Radiomics signature building

A total of 1037 radiomics features on NC, AP and VP images were exacted for each tumor. After rigorous feature screening, 3 NC features, 4 AP features and 3 VP features were selected as the final signatures for differentiating paragangliomas and schwannomas. The feature names and distributions are listed in Table 2. Following stepwise regression analysis, six features were removed after combining the radiomics features of NC, AP and VP phases.

**Table 2:** The final signatures selected from 3D radiomics features.

No contrast enhancement^3^	Arterial phase^4^	Venous phase^3^	Radiomics^4^
NC_wavelet.HLH_firstorder_Mean	A_log.sigma.5.0.mm.3D_ firstorder _Kurtosis	V_original_ firstorder_10Percentile	A_wavelet.HLL_glszm_SmallAreaEmphasis
NC_wavelet.LLL_firstorder_10Percentile	A_wavelet.LHL_ glszm_GrayLevelNonUniformityNormalized	V_original_glcm_JointEnergy	V_original_ firstorder_10Percentile
NC_wavelet.LLL_glcm_Imc2	A_wavelet.HLL_glszm_SmallAreaEmphasis	V_wavelet.LLL_ glrlm_RunEntropy	V_original_glcm_JointEnergy
	A_wavelet.LLL_ firstorder_10Percentile		V_wavelet.LLL_ glrlm_RunEntropy

### Discriminative performance of the radiomics model

Four models were built based on the above radiomics signatures for preoperatively differentiating paragangliomas and schwannomas. The AUC, accuracy, sensitivity, specificity, PPV and NPV are listed in Table 3. As shown in Fig. 3A,B and Table 3, the discriminative AUCs of the NC, AP and VP model were 0.826, 0.892 and 0.969 in the training cohort, 0.744, 0.904 and 0.929 in the internal validation cohort and 0.664, 0.703 and 0.748 in the external validation cohort, respectively. In addition, we combined the radiomics signatures of the NC, AP, and VP phases into a Radiomics model and it had a higher predictive AUC than the other radiomics model (Fig. 3A,B, Table 3). The AUC values of the Radiomics model were 0.977 (95% CI 0.947–1.000) in the training cohort, 0.936 (95% CI 0.840–1.000) in the internal validation cohort and 0.832 (95% CI 0.655–1.000) in the external validation cohort. The accuracy, sensitivity and specificity were 0.937, 0.909 and 0.967 in the training cohort, 0.920, 0.923 and 0.917 in the internal validation cohort and 0.875, 0.846 and 0.818 in the external validation cohort, respectively (Tables 3, 4).

**Table 3:** Discriminative performance of different models in training and internal validation cohorts.

Feature_num	Methods	Training cohort						Internal validation cohort					
		AUC	Accuracy	Sensitivity	Specificity	PPV	NPV	AUC	Accuracy	Sensitivity	Specificity	PPV	NPV
3	NC	0.826 (0.721–0.931)	0.778 (0.655–0.873)	0.909 (0.576–1.000)	0.633 (0.433–0.800)	0.732 (0.633–0.750)	0.864 (0.812–0.889)	0.744 (0.542–0.946)	0.720 (0.506–0.879)	0.692 (0.154–0.923)	0.750 (0.248–1.000)	0.750 (0.400–0.800)	0.692 (0.427–0.750)
4	AP	0.892 (0.814–0.970)	0.841 (0.727–0.921)	0.909 (0.605–1.000)	0.767 (0.300–0.900)	0.811 (0.741–0.825)	0.885 (0.750–0.900)	0.904 (0.764–1.000)	0.840 (0.639–0.955)	0.846 (0.231–1.000)	0.833 (0.583–1.000)	0.846 (0.600–0.867)	0.833 (0.778–0.857)
3	VP	0.969 (0.925–1.000)	0.968 (0.890–0.996)	0.939 (0.848–1.000)	1.000 (0.399–1.000)	1.000 (1.000–1.000)	0.938 (0.857–0.938)	0.929 (0.824–1.000)	0.880 (0.688–0.975)	0.923 (0.462–1.000)	0.833 (0.417–1.000)	0.857 (0.750–0.867)	0.909 (0.833–0.923)
4	Radiomics	0.977 (0.947–1.000)	0.937 (0.845–0.982)	0.909 (0.787–1.000)	0.967 (0.732–1.000)	0.968 (0.963–0.971)	0.906 (0.880–0.909)	0.936 (0.840–1.000)	0.920 (0.740–0.990)	0.923 (0.308–1.000)	0.917 (0.333–1.000)	0.923 (0.800–0.929)	0.917 (0.800–0.923)
2	Clinical	0.856 (0.763–0.948)	0.810 (0.691–0.898)	0.667 (0.333–0.818)	0.967 (0.667–1.000)	0.957 (0.917–0.964)	0.725 (0.645–0.732)	0.750 (0.552–0.948)	0.760 (0.549–0.906)	0.615 (0.154–0.848)	0.917 (0.331–1.000)	0.889 (0.667–0.917)	0.688 (0.443–0.706)
3	Combined	0.984 (0.952–1.000)	0.984 (0.915–1.000)	0.970 (0.909–1.000)	1.000 (0.399–1.000)	1.000 (1.000–1.000)	0.968 (0.923–0.968)	0.955 (0.864–1.000)	0.960 (0.796–0.999)	1.000 (0.308–1.000)	0.917 (0.750–1.000)	0.929 (0.800–0.929)	1.000 (1.000–1.000)

AP, radiomics model of arterial phase; Clinical, fusion of clinical and CT characteristics; Combined, fusion of Radiomics model and clinical model; VP, radiomics model of venous phase; NC, radiomics model of no contrast enhancement; Radiomics, fusion of radiomics features of NC, AP, and V.

*AP* arterial phase, *AUC* area under the curve, *NC* no contrast enhancement, *NPV* negative predictive value, *PPV* positive predictive value, *VP* venous phase.

**Table 4:** Discriminative performance of different models in external validation cohort.

Methods	AUC	Accuracy	Sensitivity	Specificity
NC	0.664	0.667	0.692	0.636
AP	0.703	0.750	0.769	0.636
VP	0.748	0.750	0.769	0.727
Radiomics	0.832	0.875	0.846	0.818
Clinical	0.787	0.791	0.846	0.727
Combined	0.871	0.917	0.923	0.818

AP, radiomics model of arterial phase; Clinical, fusion of clinical and CT characteristics; Combined, fusion of Radiomics model and clinical model; VP, radiomics model of venous phase; NC, radiomics model of no contrast enhancement; Radiomics, fusion of radiomics features of NC, AP, and VP.

*AP* arterial phase, *AUC* area under the curve, *NC* no contrast enhancement, *VP* venous phase.

### Discriminative performance of the combined model

As shown in Tables 3, 4 and Fig. 3, we developed a clinical-radiomics combined model incorporating three CT characteristics (enhancement magnitude at AP and VP) and four radiomics signatures. The clinical-radiomics combined model showed superior predictive performance for differentiating paragangliomas and schwannomas compared to either the clinical model or the radiomics models alone; the AUC values of the clinical-radiomics combined model were 0.984 (95% CI 0.952–1.000) in the training cohort, 0.955 (95% CI 0.864–1.000) in the internal validation cohort and 0.871 (95% CI 0.710–1.000) in the external validation cohort. The accuracy, sensitivity and specificity were 0.984, 0.970 and 1.000 in the training cohort, 0.960, 1.000 and 0.917 in the internal validation cohort and 0.917, 0.923 and 0.818 in the external validation cohort, respectively. Based on the results of Delong test, the performance of the clinical-radiomics combined model was significantly better than the NC model and AP model (*P* < 0.05) in the training cohort.

**Figure 4:**
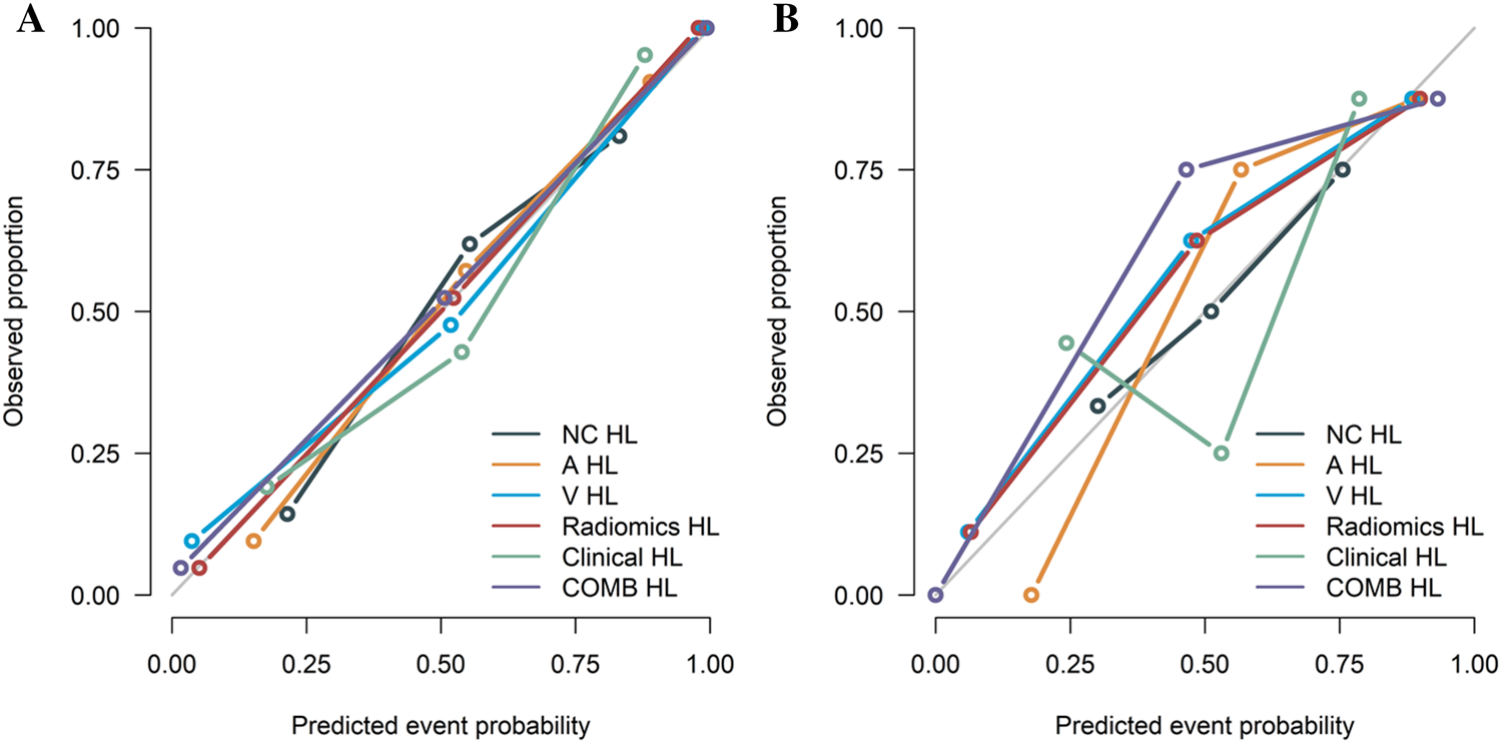
Calibration curves of the different models in training (**A**) and internal validation cohorts (**B**). *A* radiomics model of arterial phase, *COMB* Radiomics model and clinical model, *D* radiomics model of delayed phase, *Radiomics* radiomics model of fusion of arterial phase, delayed phase and venous phase features, *V* radiomics model of venous phase.

**Figure 5:**
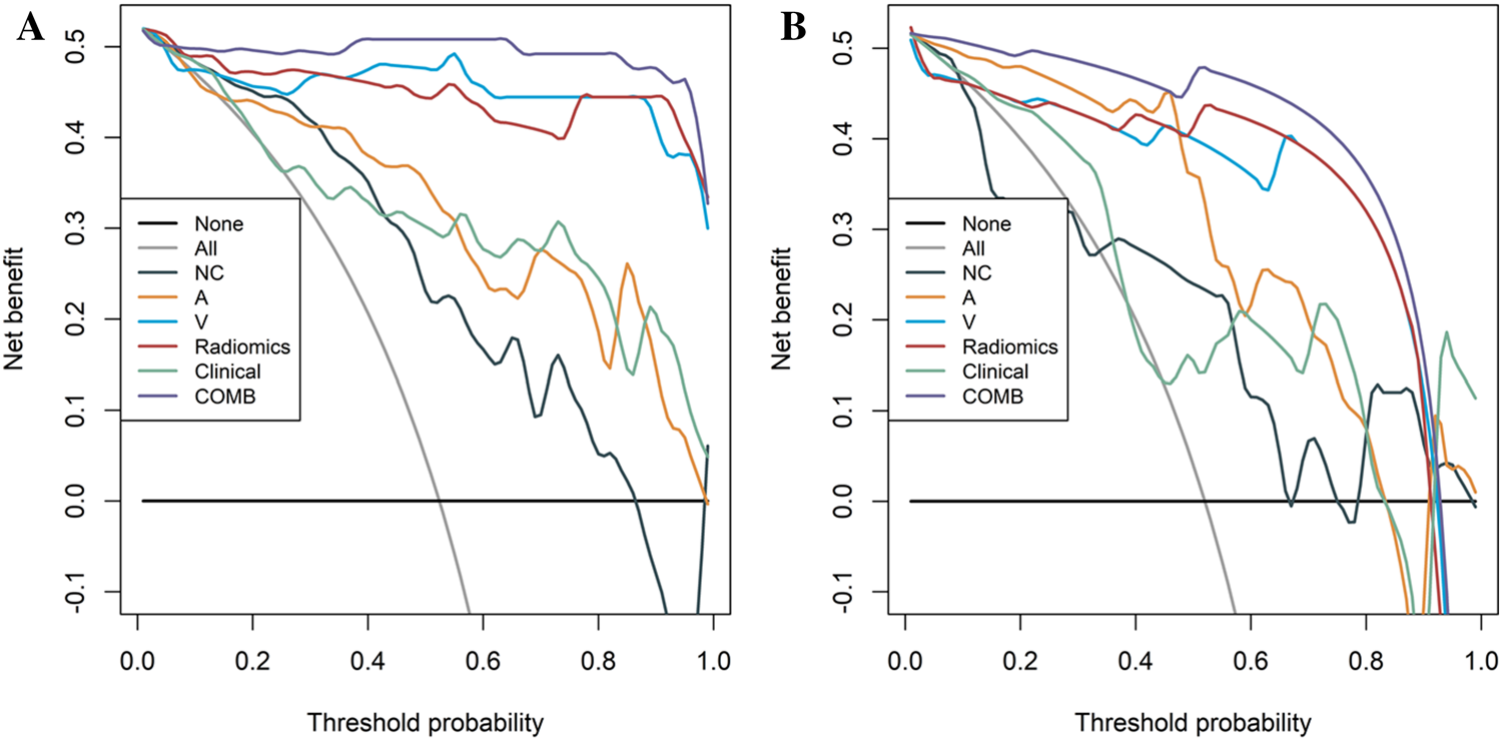
Decision curve analysis of different models in training (**A**) and internal validation cohorts (**B**). *A* radiomics model of arterial phase, *COMB* Radiomics model and clinical model, *D* radiomics model of delayed phase, *Radiomics* radiomics model of fusion of arterial phase, delayed phase and venous phase features, *V* radiomics model of venous phase.

As shown in Fig. 4A,B, the calibration curve of the different models showed favourable agreement between prediction and observation in differentiating paragangliomas and schwannomas in the training cohort. The decision curve analysis for the different models is presented in Fig. 5A,B. The clinical-radiomics combined model achieved more clinical utility in differentiating paragangliomas and schwannomas than other radiomics models or clinical model in the training cohort and internal validation cohort.

### Comparation of the diagnostic accuracy between different models and radiologists

Diagnostic accuracy of different models and radiologists for pheochromocytomas and schwannomas was presentation in Table 5. Four radiomics models, clinical model and clinical-radiomics combined model had a higher diagnostic accuracy for pheochromocytomas than the two radiologists. As for schwannomas, AP, VP, Radiomics, clinical and clinical-radiomics combined models had a higher diagnostic accuracy for schwannomas than the two radiologists.

**Table 5:** Comparation of the diagnostic accuracy in paragangliomas and schwannomas between radiomics models and radiologists.

Methods	Paragangliomas (n = 46)	Schwannomas (n = 42)
	Accuracy	Accuracy
NC	0.848	0.667
AP	0.891	0.786
VP	0.913	0.952
Radiomics	0.913	0.952
Clinical	0.869	0.881
Combined	0.956	0.928
Radiologist 1	0.761	0.785
Radiologist 2	0.826	0.762

AP, radiomics model of arterial phase; Clinical, fusion of clinical and CT characteristics; Combined, fusion of Radiomics model and clinical model; VP, radiomics model of venous phase; NC, radiomics model of no contrast enhancement; Radiomics, fusion of radiomics features of NC, AP, and VP.

*AP* arterial phase, *NC* no contrast enhancement, *VP* venous phase.

## Discussion

Pheochromocytomas can cause symptoms such as episodic hypertension, tachycardia and diaphoresis due to catecholamine release, these clinical manifestations occur in only a fraction of patients, and nearly half of tumors are non-functional and potentially functional. It is difficult for differentiating non-functional and potentially functional pheochromocytomas from other retroperitoneal masses, especially schwannomas. Ultrasound- or CT-guided percutaneous paraganglioma biopsy can be used for the diagnosis of pheochromocytoma. However, hypersecretion of catecholamines during biopsy in functional paraganglioma can also have serious consequences, such as hypertensive crisis^17^. Therefore, it is of great clinical value to develop a non-invasive method to accurately identify pheochromocytoma prior to surgery.

In this study, we developed radiomics, clinical and clinical-radiomics combined models for the preoperative differentiation of the paragangliomas and schwannomas. Our study showed that the NC, AP, VP, Radiomics and clinical models had encouraging differentiation performance. Additionally, the clinical-radiomics combined model that combined NC, AP, VP radiomics features and clinical characteristics had a higher outstanding performance than other models in both the training, internal validation and external validation cohorts. The calibration curve and decision curve of clinical-radiomics combined model showed excellent stability and actual benefit.

In our study, 1037 quantitative features were extracted from the CT images to build radiomics signatures. During the image preprocessing stage, the LoG filter and wavelet filter were applied to process the original image. Among the NC, AP and VP radiomics signatures, the signatures related to the wavelet filter accounted for 7/10. This indicates that wavelet filter is very important for the extraction of features related to differentiate pheochromocytomas from schwannomas. In this study, 5 texture features and 5 first-order features were found to be radiomics signatures to discriminate the two tumors. Radiomics quantifies not only morphological features (such as size and edge) but also internal texture features that are not visible to the human eye. Previous studies have proved that radiomics features can represent intra-tumor heterogeneity, which has attracted more and more attention in recent years^18,19^.

Among the NC, AP and VP models for differentiation of the two tumors, the VP models showed the highest performance. The possible explanation is that the internal structure of the tumor is different at four CT phases. The two types of tumors also showed different characteristics in multiphase contrast-enhanced CT images. Most paragangliomas show a "fast-in-fast-out" enhancement pattern, that is, obvious enhancement in the arterial phase and venous phases and a gradual decrease in the delayed phases^20^. The majority of schwannomas presented as a progressive enhancement mode, with no or mild enhancement in the arterial phase and a gradual increase in the venous phase and delayed phases^21^. Previous literature showed that the increase in structure in the enhanced image was proportional to the iodine concentration^22^. The iodine concentration in the VP images was higher than that in the AP and NC images, so the VP image contains more image information, which may be a rational explanation for the higher predictive performance in the VP model.

There are some limitations to our study. First, the sample size of this study is small. Second, reproducibility should be further addressed in future studies in multi-center with large sample size. Third, although manual delineation seems the most intuitive and easily implemented way of obtaining a target volume. Nevertheless, manual segmentation is strongly operator-dependent, producing inter- and intra-observer variability results. In addition, manual segmentation is labor-intensive, time-consuming^23,24^. To reduce the operator interaction in the segmentation process and to improve the reproducibility of radiomics studies, automatic or semi-automatic software platforms (such as 3D Slicer, LifEx, etc…) should be used in a future study^25^. Finally, Feature harmonization could be adopted and added in our future study to overcome batch effects generated by different imaging devices.

In conclusion, CT radiomics had the excellent differentiation performance between retroperitoneal paragangliomas and schwannomas, which may contribute to the development of individualised therapeutic strategies prior to surgery.

## Data Availability

Data generated or analyzed during the study are available from the corresponding author by request.

## Abbreviations

AP: Arterial phase
AUC: Area under curve
CI: Confidence interval
CT: Computed tomography
DCA: Decision curve analysis
NC: No contrast enhancement
NPV: Negative predictive value
PPV: Positive predictive value
ROC: Receiver operating characteristic curve
VP: Venous phase
